# Effect of previous administration of potassium iodine and different durations of low iodine diets for radioiodine therapy on the treatment of Graves' disease in iodine-rich areas

**DOI:** 10.1007/s00259-023-06523-7

**Published:** 2023-11-27

**Authors:** Mika Tamura, Kunihiro Nakada, Haruna Iwanaga, Naotoshi Fujita, Katsuhiko Kato

**Affiliations:** 1 Department of Radiological and Medical Laboratory Sciences, Nagoya University Graduate School of Medicine, Nagoya, Japan; 2 Department of Clinical Nutrition, Hokko Memorial Hospital, Sapporo, Japan; 3 Department of Radiology, Hokko Memorial Hospital, Sapporo, Japan; 4 Department of Radiological Technology, Nagoya University Hospital, Nagoya, Japan; 5 Functional Medical Imaging, Biomedical Imaging Sciences, Division of Advanced Information Health Sciences, Department of Integrated Health Sciences, Nagoya University Graduate School of Medicine, 1-20, Daikominami 1-Chome, Higashi-Ku, Nagoya, 461-8673 Japan

**Keywords:** Radioiodine therapy, Low iodine diet, Graves’ hyperthyroidism, Urinary iodine concentration, Iodine-rich areas

## Abstract

**Purpose:**

To examine whether adherence to a low-iodine diet (LID) enhances the therapeutic efficacy of radioiodine therapy (RAI) in Graves’ hyperthyroidism (GH) in iodine-rich areas.

**Methods:**

We retrospectively evaluated 185 patients with GH from Aichi (n = 114) and Hokkaido (n = 71) Prefectures. Patients aged ≥ 18 years with GH who underwent RAI between December 2012 and March 2022 were divided into subgroups based on pretreatment with anti-thyroid drug (ATD) or potassium iodide (KI). Patients were followed up with LID from 18 days (group A) or 7 days (group H) before RAI to 3 days after RAI. The dose of radioactive iodine 131 (^131^I) was adjusted to deliver > 100 Gy to the thyroid. The associations between urinary iodine concentration on UIC2 vs. 24hRU and UIC2 vs. the 1-year RAI success rate (SR) were investigated.

**Results:**

Compared with UIC1, UIC2 was significantly decreased in all subgroups (P < 0.01). An inverse correlation between UIC2 and 24hRU was observed in the four groups; however, the difference was insignificant. The SR in groups A and H was 85% and 89%, respectively. Univariate analysis revealed no association between UIC2 and SR in each group. Additionally, stratification of the 185 patients into quartiles using UIC2 yielded no significant differences in SR (*p* = 0.79).

**Conclusions:**

LID sufficiently reduced UIC in patients undergoing RAI. Although a lower UIC2 may increase 24hRU, it did not increase the success of RAI. The benefit of LID in enhancing the efficacy of RAI in GH treatment remains uncertain.

## Introduction

Graves’ hyperthyroidism (GH) is the most common cause of hyperthyroidism. Anti-thyroid drugs (ATDs), radioactive iodine ^131^I therapy (RAI), and thyroidectomy are the main treatments for GH [1]. Serious side effects can occur following administration of ATDs and thyroidectomy. RAI is an established and safe treatment option for GH. Identifying the factors that affect RAI efficacy can help improve patient outcomes and reduce healthcare costs associated with GH. These factors include thyroid weight and hormone levels before treatment, duration of ATD use, and other clinical factors [2–4]. Two preparations are recommended before ^131^I administration to enhance RAI efficacy: withdrawal of ATDs, including methimazole (MMI), propylthiouracil (PTU), and/or inorganic iodides, such as potassium iodide (KI) [1, 5–7], and restricting iodine-containing foods, which may impair ^131^I uptake in the thyroid. Adherence to a low-iodine diet (LID) has been proposed for this purpose. Published guidelines for RAI recommend a LID for 1 − 3 weeks for postsurgical differentiated thyroid cancer [8–10]. A link between urinary iodine concentration (UIC) and RAI efficacy has been suggested in remnant tissue ablation in differentiated thyroid cancer [11–13]. However, the clinical value of LID in RAI for GH treatment remains unclear in iodine-rich areas. Few studies have investigated the relationship between UIC after LID and RAI outcomes in GH. These studies concluded that LID did not improve the success of RAI [14–16]. Thus, we aimed to evaluate the changes in UIC before and after LID in two patient groups with different backgrounds of dietary iodine intake who followed different LID regimens. Furthermore, we evaluated the association between the UIC on the day of RAI and the therapeutic efficacy of RAI in these patient groups.

## Materials and methods

### Patients

This retrospective study was approved by the local ethics committees of Nagoya University Hospital (Nagoya, Japan) (2020–0052) and Hokko Memorial Hospital (Sapporo, Japan) (I-03). Overall, 185 adults (male 25, female 160; average age 46.7 years) at two institutes in Japan were included: 114 who lived in the Aichi or Mie Prefecture and underwent RAI at Nagoya University Hospital, Nagoya, between April 2017 and March 2022 (group A), and 71 from the Hokkaido Prefecture who underwent RAI at Hokko Memorial Hospital, Sapporo, between December 2012 and May 2017 (group H). None of the patients dropped out in the year after RAI. All patients were pretreated with ATDs (MMI, PTU, and/or KI) before RAI. The patient inclusion criteria were as follows: (1) biochemically confirmed GH with positive thyrotropin receptor antibody (TRAb); (2) an estimated thyroid weight < 100 g; and (3) follow-up duration of thyroid function of > 1 year after RAI. These criteria ensured no bias between the two hospitals and that patients would be treated with the first RAI. All patients provided written informed consent to undergo RAI and associated procedures, including adherence to the LID. The sociophysiological characteristics of the patients are summarised in Table 1.

**Table 1:** Characteristics of patients with Graves’ hyperthyroidism

	Total	Group	
		A	H
Patients^a^	185	114 (61.6%)	71 (38.4%)
Gender^a^ (M/F)	25/160	13/101	12/59
Residence^a^ (inland/seashore)	88/97	42/72	46/25
Anti-thyroid medication (without KI/with KI)	109/76	74/40	35/36
BMI^b^ (kg/m^2^)	22.7 (19.8–25.2)	22.6 (19.5–25.5)	23.0 (20.9–24.8)
e-GFR^b^ (mL/min/1.73m^2^)	101.3 (80.4–116.0)	102.9 (75.5–118.0)	99.2 (83.4–112.0)
Age^b^ (years)	46.7 (34.0–57.0)	46.5 (35.0–56.0)	46.9 (33.5–58.5)

^a^Figures show numbers. ^b^Figures show average values and IQR (Interquartile range)

Group A: Patients from the Nagoya University Hospital living in Aichi. Group H: Patients from the Hokko Memorial Hospital living in Hokkaido. KI, potassium iodide; BMI, body mass index; e-GFR, estimated glomerular filtration rate

### Patient preparation and LID for RAI

**Fig. 1:**
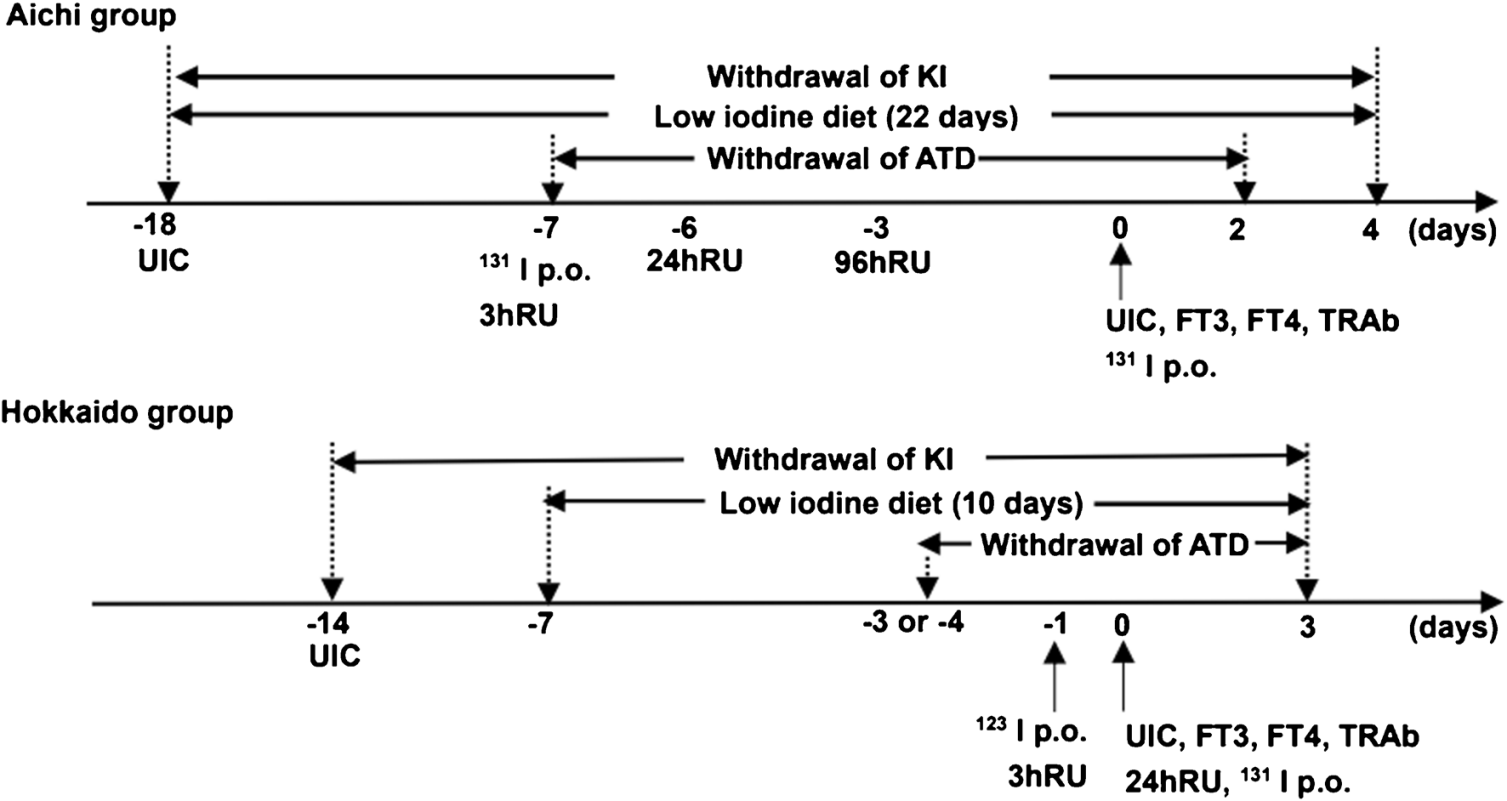
Protocol for radioactive iodine treatment at the two facilities. Aichi group: Patients in Nagoya University Hospital living in Aichi. ATD, antithyroid drug; FT3: free triiodothyronine; FT4: free thyroxine; Hokkaido Group: Hokko Memorial Hospital, Hokkaido; KI: potassium iodine; p.o.: per os; RU: radioiodine uptake; TRAb: thyrotropin receptor antibody; UIC: urinary iodine concentration

Two preparatory regimens were used (Fig. 1). In group A, pretreatment with KI was withdrawn 18 days before RAI, whereas ATDs were discontinued 7 days before RAI. In group H, KI was withdrawn 14 days before RAI, while ATDs were discontinued 3–4 days before RAI. After nutritional counselling with a dietitian or physician, patients in group A underwent LID from 18 days before RAI to 4 days after RAI. Patients in group H underwent LID from 7 days before to 3 days after RAI. We developed a brochure for patients who were followed up for a LID with RAI [17]. This was used for counselling in group H. In group A, commercially available materials were used for counselling.

### RAI

The dose of ^131^I was determined according to Marinelli’s formula [18]. In this formula, the weight of the thyroid gland (TW), 24-h radioiodine uptake (24hRU), and the effective half-life of radioiodine in the thyroid (T1/2eff) served as the variables for the administered dose of ^131^I. In group A, radioiodine uptake (RU) was measured using ^131^I and an uptake probe (thyroid uptake system: AZ-800, Anzai Medical, Tokyo, Japan). Exactly 3.7 MBq of ^131^I was administrated 7 days before RAI. RU was sequentially measured at 3, 24, and 96 h to determine the T1/2eff. In group H, RU was determined using ^123^I and a gamma camera (Millennium MG, GE Healthcare, Milwaukee, USA), and 7.4 MBq of ^123^I was administered the day before RAI to perform thyroid scintigraphy and determine the 24hRU. 24hRU determined using ^123^I based on a standard method, a region of interest (ROI) was drawn around the margins of the thyroid gland and ROI in the supraclavicular area was used for background subtraction. The whole-body count was measured using a scintillation survey meter at 24, 72, 96, and 120 h after RAI in seven patients. The average value of 5.7 days from these patients was applied to the other patients to calculate the dosage of ^131^I. In group A, TW was measured using computed tomography (CT) (Aquilion 64 or Aquilion PRIME SP; Canon Medical Systems, Tokyo, Japan). The patients underwent non-enhanced neck CT examinations for thyroid volumetry. Contour extraction of the thyroid from CT images (5-mm slice thickness) was manually performed by a single radiologist (nuclear medicine physician). The sum of thyroid volumes obtained from each slice was denoted as the total thyroid volume. In group H, TW was measured using a Aquilion PRIME CT scanner (Canon Medical Systems) or by ultrasonography (US) (Xario; Canon Medical Systems). Thyroid volume was obtained by US according to the following formula:

$$\mathrm\pi/6\times\mathrm{long}\times\mathrm{short}\times\mathrm{horizontal}\;\mathrm{diameter}$$ [4]

### Laboratory tests

Thyroid function (free tri-iodothyronine [FT3], free thyroxine [FT4], and thyroid-stimulating hormone [TSH]) and TRAb levels were measured on the day of RAI. The UIC was measured twice using spot urine samples before commencing the LID (UIC1) and on the day of RAI (UIC2). In group A, thyroid function was determined using a CLEA kit (Abbott Diagnostic Medical, Tokyo, Japan; normal range: FT3 1.68–3.67 pg/mL, FT4 0.70–1.48 ng/dL, TSH 0.35–4.94 μIU/mL). In group H, thyroid function was evaluated using the ECLEA (ECLEA) Kit (Roche Diagnostic Medical, Tokyo, Japan; normal range: FT3 2.3 − 4.0 pg/mL, FT4 0.90–1.7 ng/dL, TSH 0.50–5.00 μIU/mL). TRAb was determined using an ECLES Kit (Roche Diagnostic; normal range: < 2.0 IU/L in both institutes). UIC was measured by visible absorptiometry as previously described [19]. Thyroid function was monitored every 1–3 months after RAI. RAI was defined as successful when euthyroidism, subclinical hypothyroidism, or hypothyroidism was achieved within 1 year after RAI.

### Statistical analysis

We investigated the changes in UIC before and after LID. We also investigated the relationship between UIC2 vs. 24hRU (group A and group H) and UIC2 vs. T1/2eff (group A only). Additionally, we investigated whether lower UIC2 enhances RAI success rate (SR). Commercially available KI in Japan contains 38.2 mg of iodine per 50 mg of the pill. Because 76 of 185 patients had been taking 1 − 6 pills of KI, the patient groups were further classified into four subgroups based on the pretreatment use of KI: A1 (group A pretreated with ATDs without KI, n = 74); A2 (group A pretreated with ATDs combined with KI or KI alone, n = 40); H1 (group H pretreated with ATDs without KI, n = 35); and H2 (group H pretreated with ATDs combined with KI, n = 36). The 185 patients underwent further stratification into quartiles based on UIC2, regardless of the pretreatment use of KI. The Wilcoxon signed-rank test was used to investigate changes in UIC before and after LID initiation. Spearman’s rank correlation coefficient was used to investigate the relationship between UIC2 vs. 24hRU and UIC2 vs. T1/2eff (group A only). This was a non-parametric correlation assessment of the two groups. FT3, FT4, TRAb, TW, UIC2 and 24hRU may be factors affecting the efficacy of RAI, and this hypothesis was tested using Fisher’s exact test. We examined the factors influencing the treatment effects by correcting for the effects of each variable. Similarly, T1/2eff might be a success factor for RAI, and we tested this hypothesis using the Fisher’s exact test. The Fisher’s exact test was used for nominal variables and independent comparisons between groups. Statistical significance was set at p < 0.05. All statistical analyses were performed using EZR (Saitama Medical Center, Jichi Medical University, Saitama, Japan), a graphical user interface for R (The R Foundation for Statistical Computing, Vienna, Austria) [20].

## Results

There were no statistically significant differences in age, sex, body mass index, or estimated glomerular filtration rate between groups A and H (Mann–Whitney U test) (Table 1). Group H contained more patients pretreated with a combination of ATDs and KI or KI alone (p < 0.05). More patients in group A lived on the seashore than those in group H (p < 0.01) (Fisher’s exact test).

### Changes in UIC before and after LID

The clinical data for the four subgroups are shown in Table 2. The average FT3 level was higher in group H2 than in groups A1 (p < 0.01) and H1 (p < 0.01). Similarly, the mean serum FT4 level in group H2 was significantly higher than that in A1 (p < 0.01). The TW was significantly higher in group A2 than in groups A1 (p < 0.05), H1 (p < 0.01) and H2 (p < 0.01).

**Table 2:** Thyroid function, TRAb, 24hRU, and thyroid weight in the four subgroups

	Group	FT3 (pg/mL)	FT4 (ng/dL)	TRAb (IU/L)	24hRU (%)	Thyroid weight (g)
Average	A1	7.4 ± 6.6	1.7 ± 0.9	18.6 ± 15.9	64.1 ± 17.5	48.3 ± 21.9
	A2	11.6 ± 6.5	2.6 ± 1.1	10.2 ± 8.8	63.1 ± 23.5	61.1 ± 23.4
	H1	6.1 ± 5.7	2.8 ± 2.2	18.6 ± 14.3	60.7 ± 11.9	41.2 ± 24.1
	H2	12.7 ± 8.1	4.0 ± 2.5	16.2 ± 11.4	58.8 ± 16.6	43.0 ± 20.3
*p*-value	A1:A2	< 0.01	< 0.01	0.22	0.96	< 0.05
	A1:H1	0.41	0.13	0.99	0.41	0.29
	A1:H2	< 0.01	< 0.01	1.00	0.40	0.59
	A2:H1	< 0.01	0.79	0.08	0.28	< 0.01
	A2:H2	0.92	0.26	< 0.05	0.26	< 0.01
	H1:H2	< 0.01	0.09	0.99	1.00	0.88

A1: Patients in Nagoya University Hospital living in Aichi pretreated with only antithyroid drugs (ATD). A2: Patients in Nagoya University Hospital living in Aichi pretreated with ATD and potassium iodide (KI) or KI alone. H1: Patients in Hokko Memorial Hospital living in Hokkaido pretreated with only ATD. H2: Patients in Hokko Memorial Hospital living in Hokkaido pretreated with ATD and KI or KI alone. The Kruskal–Wallis test is used. FT3, Free triiodothyronine; FT4, Free thyroxine; TRAb, thyrotrophin receptor antibody; RU, radioiodine uptake

The average UIC1 vs. UIC2 in groups A1, A2, H1, and H2 are shown in Table 3. The average UIC2 in groups A1, A2, H1, and H2 were approximately 24, 0.2, 20, and 0.09% of UIC1, respectively. Exactly 88 of 114 patients (77%) in group A and 44 of 71 (65%) in group H had UIC2 values < 100 μg/gCRE. In contrast, 10 of 114 patients (9%) in group A and 12 of 71 (17%) in group H had a UIC2 > 200 μg/gCRE.

**Table 3:** Changes in UIC before and after the LID

	Group	UIC1 (µg/gCRE)	UIC2 (µg/gCRE)	*p*-value	UIC2/UIC1 (%)
Average	A1	311.7 ± 383.9	75.0 ± 113.9	< 0.01	24.1
	A2	56983.1 ± 65579.8	108.5 ± 97.9	< 0.01	0.2
	H1	488.5 ± 818.4	98.2 ± 110.1	< 0.01	20.1
	H2	187085.6 ± 240187.2	164.3 ± 253.7	< 0.01	0.09
*p*-value	A1:A2	*p* < 0.01	*p* < 0.01	–	–
	A1:H1	0.18	*p* < 0.05	–	–
	A1:H2	*p* < 0.01	*p* < 0.01	–	–
	A2:H1	*p* < 0.01	0.42	–	–
	A2:H2	*p* < 0.01	0.40	–	–
	H1:H2	*p* < 0.01	0.14	–	–
UIC < 100 (number)	A1	13	60	–	–
	A2	0	28	–	–
	H1	7	24	–	–
	H2	0	22	–	–
UIC > 200 (number)	A1	19	5	–	–
	A2	26	5	–	–
	H1	19	4	–	–
	H2	36	8	–	–

UIC1: UIC measured before initiation of LID

UIC2: UIC measured on the day of radioactive iodine ^131^I therapy

A1: Patients in Nagoya University Hospital living in Aichi pretreated with ATD alone

A2: Patients in Nagoya University Hospital living in Aichi pretreated with ATD and KI or KI alone

H1: Patients in Hokko Memorial Hospital living in Hokkaido pretreated with ATD alone

H2: Patients in Hokko Memorial Hospital living in Hokkaido pretreated with ATD and KI or KI alone

The Mann–Whitney U test is used

ATD, antithyroid drug; CRE, creatinine; KI, potassium iodide; LID, low iodine diet; UIC, urinary iodine concentration

### Association between clinical factors including UIC2 and RAI uptake

**Fig. 2:**
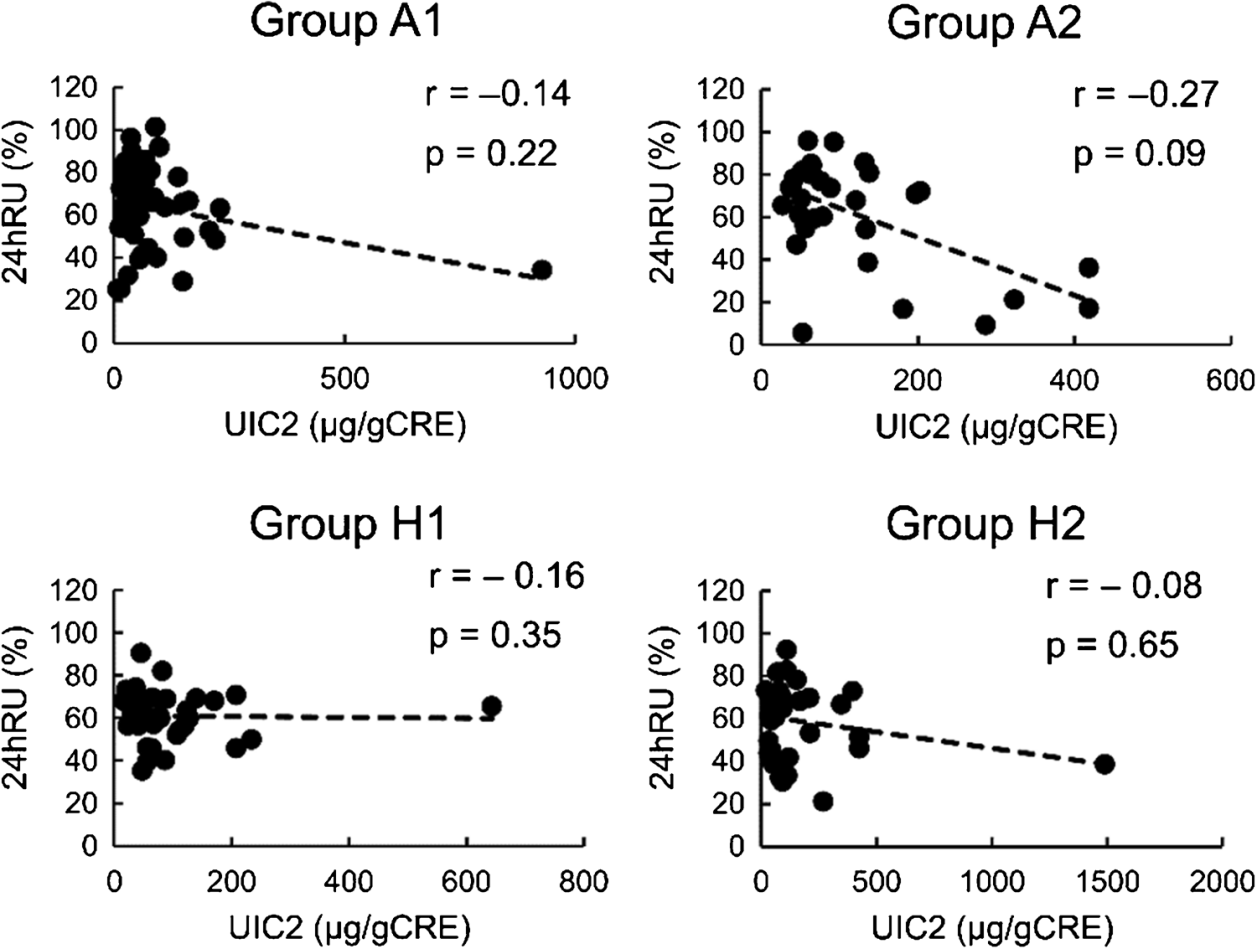
Relationship between 24 h radioiodine uptake (24hRU) and urinary iodine concentration on the day of treatment. Group A1: Patients in Nagoya University Hospital living in Aichi pretreated with an antithyroid drug (ATD) alone. Group A2: Patients in Nagoya University Hospital living in Aichi pretreated with ATD and potassium iodide (KI) or KI alone. Group H1: Patients in Hokko Memorial Hospital living in Hokkaido pretreated with ATD alone. Group H2: Patients in Hokko Memorial Hospital living in Hokkaido pretreated with ATD and KI, or KI alone. Spearman’s rank correlation test is used

**Fig. 3:**
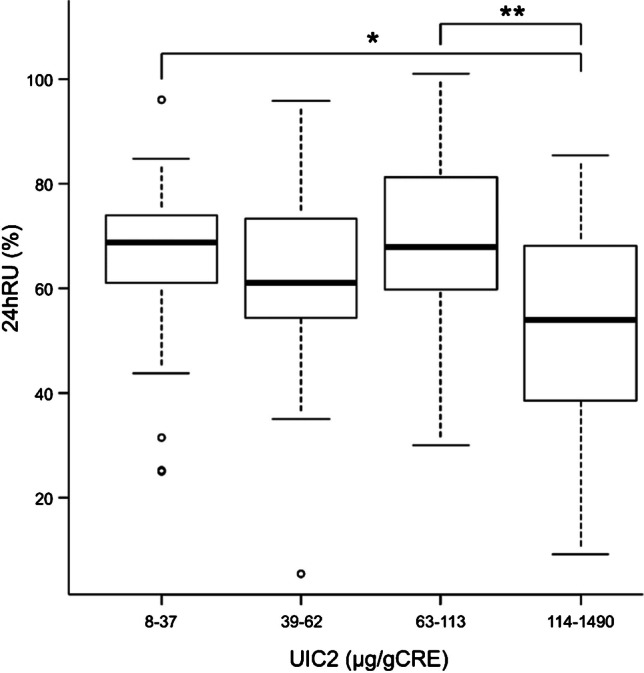
24hRU in patient groups stratified according to UIC2 quartiles *: p = 0.008; **: p = 0.005. 24hRU: 24 h radioiodine uptake. UIC2: urinary iodine concentration measured on the day of radioactive iodine 131I therapy. The Kruskal-Wallis test is used

There was no significant difference in the 24hRU among the four groups (Table 2). The correlation coefficients between UIC2 and 24hRU in A1, A2, H1, and H2 were -0.14, -0.27, -0.16, and -0.08, respectively. UIC2 was negative but not significantly correlated with 24hRU in the four groups (Fig. 2). Spearman’s rank correlation showed that FT3 and FT4 in group A1, TRAb in group H2, and TW in groups A1, H1, and H2 were positively associated with 24hRU (Table 4). In the patient groups stratified into quartiles according to UIC2, the 24hRU in the lowest quartile group (UIC 2:8–37 µg/gCRE) was significantly higher than that in the highest quartile group (UIC 2:114–1490 µg/gCRE, p < 0.01) (Fig. 3).

**Table 4:** Relationship between clinical factors and 24hRU

Group		FT3	FT4	TRAb	TW
A1	r	0.48	0.40	0.04	0.36
	p	0.01	< 0.01	0.73	< 0.01
A2	r	0.79	0.70	0.24	0.30
	p	< 0.01	< 0.01	0.13	0.06
H1	r	0.10	-0.20	-0.14	0.51
	p	0.59	0.25	0.41	< 0.01
H2	r	0.14	0.25	0.38	0.41
	p	0.42	0.14	< 0.05	< 0.05

r = correlation coefficient

A1: Patients in Nagoya University Hospital living in Aichi pretreated with ATD alone

A2: Patients in Nagoya University Hospital living in Aichi pretreated with ATD and KI or KI alone

H1: Patients in Hokko Memorial Hospital living in Hokkaido pretreated with ATD alone

H2: Patients in Hokko Memorial Hospital living in Hokkaido pretreated with ATD and KI or KI alone

ATD, ATD, antithyroid drug; FT3, Free triiodothyronine; FT4, Free thyroxine; KI, potassium iodide; TRAb, thyrotrophin receptor antibody; TW, thyroid gland; RU, radioiodine uptake; 24hRU, 24 h radioiodine uptake

Spearman’s rank correlation is used

### Association between UIC2 and effective half-life of ^131^I in the Aichi group

**Fig. 4:**
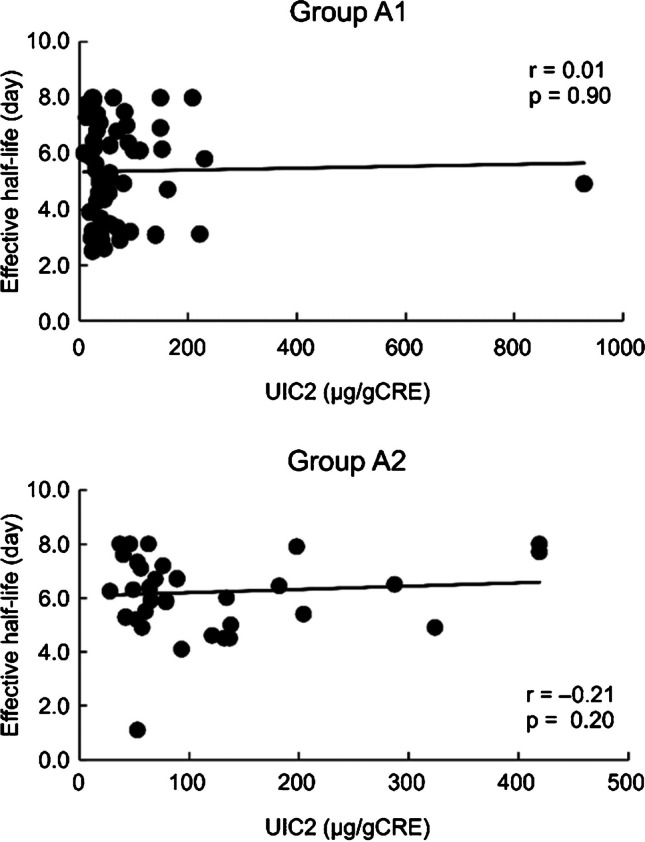
Association between UIC2 and effective half-life of ^131^I in the Aichi group. Group A1: Patients in Nagoya University Hospital living in Aichi pretreated with ATD alone. Group A2: Patients in Nagoya University Hospital living in Aichi pretreated with ATD and KI or KI alone. Spearman’s rank correlation test is used. ATD, antithyroid drug; KI, potassium iodide; UIC, urinary iodine concentration

**Fig. 5:**
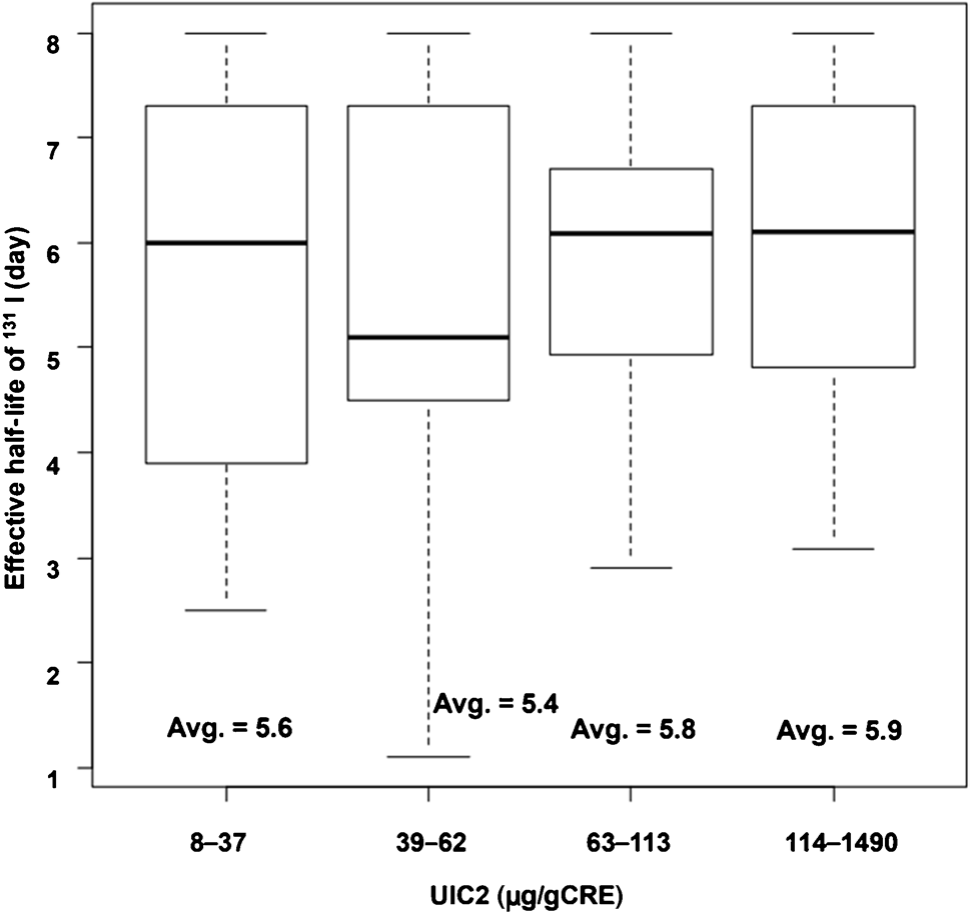
Average T1/2eff among the four patient groups stratified by UIC2 into quartiles. Avg. = average value. The Kruskal–Wallis test is used

The average T1/2eff (in days) in groups A1 and A2 were 5.4 and 6.2, respectively. The correlation coefficients between UIC2 and T1/2eff were 0.01 and -0.21 in the A1 and A2 group, respectively, which were insignificant (Fig. 4). When patients in group A were stratified according to UIC2 in quartiles, the average T1/2eff values in each quartile were 5.6, 5.4, 5.8, and 5.9, respectively. There was no significant difference in the average T1/2eff values among the four patient groups (Fig. 5).

### Association between UIC2 and 1-year SR of RAI

**Fig. 6:**
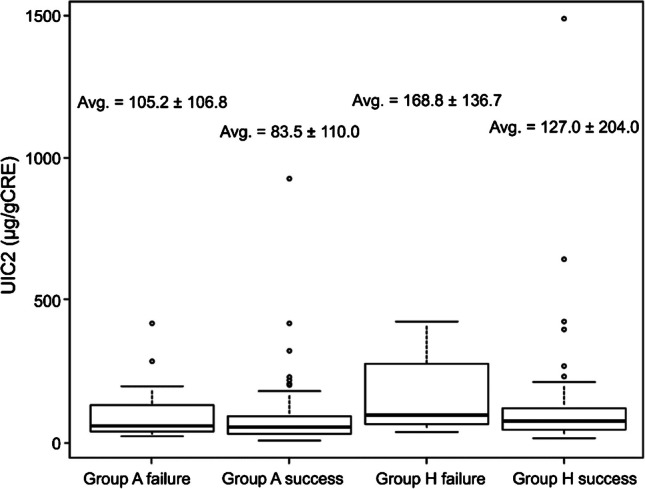
UIC2 according to RAI evaluation in groups A and H. Group A: Patients living in Aichi, Nagoya University Hospital. Group H: Patients in the Hokko Memorial Hospital living in Hokkaido. UIC2, urinary iodine concentration measured on the day of radioactive iodine I-131 µg/gCRE therapy. Success: RAI was considered successful when euthyroidism, subclinical hypothyroidism, or hypothyroidism was achieved within 1 year after RAI. Failure: Other than success. The Kruskal–Wallis test is used. Avg. = average value

The overall 1-year SR of RAI in all patients, group A, and group H, were 86.5% (160/185), 85.1% (97/114), and 88.7% (63/71), respectively. The SR of RAI in groups A and H were comparable (p = 0.52). In addition, the SR in group A1, A2, H1, and H2 were 95.9% (71/74), 65.0% (26/40), 85.6% (31/35), and 88.9% (32/36), respectively. The SR in group A2 was significantly low in the subgroup (p < 0.01). Univariate analysis using Fisher’s exact test showed that TW was the most significant factor for RAI success in both groups (Table 5). In contrast, there was no significant linkage between UIC2 and the success rate of GH. In groups A and H, the average UIC2 in patients with successful RAI was 83.5 ± 110.0 vs. 127.0 ± 204.0, respectively. There was no significant difference in the average UIC2 between patients with successful and unsuccessful RAI (Fig. 6).

**Table 5:** Univariate analysis of Fisher’s exact test results for the efficacy of radioactive iodine

Group		p	OR
A	FT3	0.29	2.02
	FT4	< 0.05	3.87
	TRAb	< 0.05	0.26
	TW	< 0.01	21.40
	UIC2	1.00	1.15
	24hRU	0.29	1.94
H	FT3	0.71	1.76
	FT4	0.48	1.82
	TRAb	< 0.05	8.52
	TW	< 0.05	8.53
	UIC2	0.15	3.46
	24hRU	1.00	0.95

A: Patients in the Aichi group

H: Patients in the Hokkaido group

FT3, free tri-iodothyronine; FT4, free thyroxine; OR: odds ratio; TRAb, thyrotrophin receptor antibody; TW, thyroid gland; UIC2, urinary iodine concentration measured on the day of radioactive iodine ^131^I therapy; 24hRU, 24 h radioiodine uptake

### Association between the effective half-life of I-131 in the Aichi group and the 1-year SR of RAI

In group A, using Fisher’s exact test, we compared the effective half-life of ^131^I with the success ratio of RAI. RAI success was significantly greater with a longer effective half-life (p = 0.02).

## Discussion

The goal of RAI in GH is the permanent treatment of thyrotoxicosis by achieving either euthyroidism or hypothyroidism. Dietary iodine intake before RAI has been considered a strategy to enhance RAI efficacy.

This retrospective study aimed to clarify the following: (1) The extent to which LID decreases the UIC; (2) Whether lower UIC is linked to a higher 24hRU or longer T1/2eff; and (3) Whether UIC on the day of RAI can function as a prognostic factor for RAI.

Dietary iodine intake may vary depending on the place of residence or the preference for broth in daily cooking. We included two patient groups with different dietary habits who underwent RAI using different dietary preparations [21, 22]. KI has gained popularity as an adjunct to ATDs in treating hyperthyroidism in Japan [23–25].

In this study, 76 of the 185 patients were pretreated with KI. This pretreatment resulted in extremely high UIC1 in groups A2 and H2 compared to those in A1 and H1 (Table 4). After adhering to a LID, UIC2 levels significantly decreased in all four subgroups. A LID for 7 and 18 days before RAI decreased iodine levels in patients with GH, regardless of their place of residence, thyroid function, TRAb, or pretreatment use of KI. However, a LID for 18 days showed no advantages over a LID for 7 days in reducing the UIC. Since LID is not a pleasant practice for patients, a relatively shorter duration of LID may be more acceptable for patients undergoing RAI.

We aimed to determine prognostic factors for the success of RAI in hyperthyroidism. Most studies have indicated TW as a significant predictive factor for RAI success [15]. In our study, TW was also shown to be the most important factor for RAI success (Table 5). In the subgroup comparison, group A2 had a significantly larger TW (Table 2) and a significantly lower SR (p < 0.01), which also indicated that TW was the most important factor for the success of RAI. However, the relationship between other clinical factors and RAI outcomes is controversial. Few studies have evaluated the effects of LID on RAI outcomes.

Another study observed no association between short-term dietary or therapeutic iodine restriction before RAI and the therapeutic effects of RAI in iodine-sufficient areas [15]. Our results are consistent with the above studies.

Increased radioiodine uptake in the thyroid or elongation of residence time should help increase the absorbed dose in the affected thyroid gland. Our study showed that a higher UIC2 might impair the determination of the therapeutic dose of ^131^I, and a lower UIC may enhance radioiodine uptake in the thyroid; however, a lower UIC does not prolong the effective half-life of ^131^I. Additionally, a higher 24hRU does not guarantee a higher success rate of RAI. RAI was successful in the patient with the highest UIC (UIC = 2) of the four subgroups. In GH, high TRAb levels function as powerful promoters of radioiodine uptake.

This study had some limitations. It was retrospective in nature. The results were obtained from patients in iodine-sufficient areas. Therefore, our findings may not apply to patients living in iodine-deficient areas. Although we did not include patients with larger goitres (> 100 g), there was considerable variance in the measured TW between the two groups. There were differences in thyroid volume calculation method (CT and US), uptake methods (thyroid uptake system and gamma camera, ^131^I and ^123^I), in groups A and H. There were also shortcomings in calculating the dosage of ^131^I. The formula used to calculate the ^131^I dose was proposed approximately 70 years ago. Although this formula is still used in our country, more sophisticated approaches have been developed [26]. Future research with larger, more representative study populations is necessary to advance RAI for patients with GH.

## Conclusion

Adhering to LID lowers UIC in patients with GH. Lower UIC can increase radioiodine uptake in the thyroid. However, a lower UIC on the day of RAI is not the only essential factor for the success of RAI in GH. Even in iodine-rich areas, stringent LID may not be necessary for RAI preparation in patients with GH.

## Data Availability

The datasets generated during and/or analysed during the current study are available from the corresponding author on reasonable request.

## Abbreviations

LID: Low-iodine diet
RAI: Radioiodine therapy
GH: Graves’ hyperthyroidism
ATD: Anti-thyroid drug
KI: Potassium iodide
SR: Success rate
MMI: Methimazole
PTU: Propylthiouracil
UIC: Urinary iodine concentration
TRAb: Thyrotropin receptor antibody
FT3: Free triiodothyronine
FT4: Free thyroxine
TSH: Thyroid-stimulating hormone
RU: Radioiodine uptake
24hRU: 24-Hour radioiodine uptake
TW: The weight of the thyroid gland
T1/2eff: The effective half-life of radioiodine in the thyroid
CT: Computed tomography
US: Ultrasonography

## References

[1] Kartamihardja AH, Massora S (2016). The influence of antithyroid drug discontinuation to the therapeutic efficacy of (131)I in hyperthyroidism. World J Nucl Med.

[2] Allahabadia A, Daykin J, Sheppard MC, Gough SC, Franklyn JA (2001). Radioiodine treatment of hyperthyroidism-prognostic factors for outcome. J Clin Endocrinol Metab.

[3] Erem C, Kandemir N, Hacihasanoglu A, Ersöz HO, Ukinc K, Kocak M (2004). Radioiodine treatment of hyperthyroidism: prognostic factors affecting outcome. Endocrine.

[4] Catargi B, Leprat F, Guyot M, Valli N, Ducassou D, Tabarin A (1999). Optimized radioiodine therapy of Graves’ disease: analysis of the delivered dose and of other possible factors affecting outcome. Eur J Endocrinol.

[5] Andrade VA, Gross JL, Maia AL (2001). The effect of methimazole pretreatment on the efficacy of radioactive iodine therapy in Graves’ hyperthyroidism: one-year follow-up of a prospective, randomized study. J Clin Endocrinol Metab.

[6] Pirnat E, Zaletel K, Gaberšček S, Hojker S (2011). The outcome of 131I treatment in Graves’ patients pretreated or not with methimazole. Hell J Nucl Med.

[7] Sato S, Noh JY, Sato S (2015). Comparison of efficacy and adverse effects between methimazole 15 mg+inorganic iodine 38 mg/day and methimazole 30 mg/day as initial therapy for Graves’ disease patients with moderate to severe hyperthyroidism. Thyroid.

[8] Silberstein EB, Alavi A, Balon HR (2012). The SNMMI practice guideline for therapy of thyroid disease with 131I 3.0. J Nucl Med.

[9] Pacini F, Schlumberger M, Dralle H, Elisei R, Smit JW, Wiersinga W (2006). European consensus for the management of patients with differentiated thyroid carcinoma of the follicular epithelium. Eur J Endocrinol.

[10] Ross DS, Burch HB, Cooper DS (2016). 2016 American Thyroid Association guidelines for diagnosis and management of hyperthyroidism and other causes of thyrotoxicosis. Thyroid.

[11] Lee M, Lee YK, Jeon TJ (2014). Low iodine diet for one week is sufficient for adequate preparation of high dose radioactive iodine ablation therapy of differentiated thyroid cancer patients in iodine-rich areas. Thyroid.

[12] Sohn SY, Choi JY, Jang HW (2013). Association between excessive urinary iodine excretion and failure of radioactive iodine thyroid ablation in patients with papillary thyroid cancer. Thyroid.

[13] Ju DL, Park YJ, Paik HY, Song Y (2015). The impact of low adherence to the low-iodine diet on the efficacy of the radioactive iodine ablation therapy. Clin Nutr Res.

[14] Santarosa VA, Orlandi DM, Fiorin LB (2015). Low iodine diet does not improve the efficacy of radioiodine for the treatment of Graves’ disease. Arch Endocrinol Metab.

[15] Nishio R, Uchida T, Suzuki L (2021). Influence of short-term dietary and therapeutic iodine restriction on the therapeutic effects of radioactive iodine therapy in patients with Graves’ disease. Thyroid.

[16] Moon JH, Yi KH (2013). The diagnosis and management of hyperthyroidism in Korea: consensus report of the Korean Thyroid Association. Endocrinol Metab (Seoul).

[17] Tamura M, Nakada K, Tsuruhara R (2016). Efficacy of dietitian-instructed low iodine diet for radioiodine remnant tissue ablation for thyroid cancer. Kaku Igaku.

[18] Marinelli LD, Quimby EH, Hine GJ (1948). Dosage determination with radioactive isotopes; practical considerations in therapy and protection. Am J Roentgenol Radium Ther.

[19] Ohashi T, Yamaki M, Pandav CS, Karmarkar MG, Irie M (2000). Simple microplate method for determination of urinary iodine. Clin Chem.

[20] Kanda Y (2013). Investigation of the freely available easy-to-use software ‘EZR’ for medical statistics. Bone Marrow Transplant.

[21] Fuse Y, Ito Y, Shishiba Y, Irie M (2022). Current iodine status in Japan: A cross–sectional nationwide survey of schoolchildren, 2014–2019. J Clin Endocrinol Metab.

[22] Zava TT, Zava DT (2011). Assessment of Japanese iodine intake based on seaweed consumption in Japan: A literature-based analysis. Thyroid Res.

[23] Okamura K, Sato K, Fujikawa M, Bandai S, Ikenoue H, Kitazono T (2014). Remission after potassium iodide therapy in patients with Graves’ hyperthyroidism exhibiting thionamide-associated side effects. J Clin Endocrinol Metab.

[24] Yoshihara A, Noh JY, Watanabe N (2015). Substituting potassium iodide for methimazole as the treatment for Graves’ disease during the first trimester may reduce the incidence of congenital anomalies: A retrospective study at a single medical institution in Japan. Thyroid.

[25] Uchida T, Goto H, Kasai T (2014). Therapeutic effectiveness of potassium iodine in drug-naïve patients with Graves’ disease: a single-center experience. Endocrine.

[26] Takata K, Amino N, Kubota S (2010). Benefit of short-term iodide supplementation to antithyroid drug treatment of thyrotoxicosis due to Graves’ disease. Clin Endocrinol (Oxf).

